# Whole-Genome Sequences of Two Pseudomonas fluorescens Strains Isolated from Roots of Tomato and Cucumber Plants

**DOI:** 10.1128/MRA.00974-18

**Published:** 2018-09-06

**Authors:** Joseph L. Sevigny, Jessica LaJoie, Shania Shehata, Elijah Christensen, Scott Cornfield, Lori Koziol

**Affiliations:** a Hubbard Center for Genome Studies, University of New Hampshire, Durham, New Hampshire, USA; b Department of Biology, New England College, Henniker, New Hampshire, USA; University of Maryland School of Medicine

## Abstract

Pseudomonas fluorescens strain EC1 was isolated from Cucumis sativus (cucumber) roots, and P. fluorescens SC1 was isolated from Solanum lycopersicum (tomato) roots. The P. fluorescens SC1 genome has a total sequence length of 6,157,842 bp, and the P. fluorescens EC1 genome has a total sequence length of 6,125,428 bp.

## ANNOUNCEMENT

Pseudomonas fluorescens is a Gram-negative, motile, and obligate aerobe that forms biofilms in the soil and on the roots of plants (1). Fluorescent pseudomonads are considered nonpathogenic to humans, and P. fluorescens has served as an important model organism for studying biofilm formation (2). Initial biofilm studies showed that P. fluorescens strain EC1 and P. fluorescens SC1 formed biofilms thicker than that of our lab strain, P. fluorescens Pf0-1. Therefore, we chose to sequence P. fluorescens EC1 and SC1 so that we may investigate the genes associated with biofilms in future studies.

*Pseudomonas* strains were isolated by suspending approximately 2.5 g of the soil sample from the rhizosphere of a tomato or cucumber root in 10 ml of phosphate-buffered saline (PBS). Dilutions were made in the PBS and plated on King’s B medium agar plates (3) and incubated for 48 h at 28°C. The plates were then placed on a UV light to detect fluorescence. Fluorescent colonies were selected and grown overnight in liquid lysogeny broth (LB), and genomic DNA was isolated using the Wizard SV genomic DNA purification system (Promega, Madison, WI). The whole-genome sequencing of P. fluorescens EC1 and SC1 was performed at the Hubbard Center for Genome Studies (University of New Hampshire, Durham, NH). After genomic DNA was isolated, a paired-end library was constructed using an Illumina Nextera DNA library preparation kit and sequenced with an Illumina HiSeq 2500 instrument for 250 cycles. The total numbers of reads for the EC1 and SC1 libraries were 5,877,526 and 4,605,954, respectively. FastQ data were trimmed for Nextera adapters and low-quality bases using Trimmomatic version 0.32 (4). For read trimming, trailing and leading bases were removed if the quality was below 3. In addition, the reads were scanned using a 4-base sliding window and trimmed if the average quality dropped below 15. Trimmed sequencing reads were then assembled using the SPAdes pipeline version 3.5 (5) with default settings. QUAST version 4.6.0 (6) was used to assess the contiguity of the assemblies, and coverage statistics were calculated by mapping FastQ reads to the assembled contigs with the Burrows-Wheeler Aligner Maximal Exact Matches (BWA-MEM) algorithm (default settings). The draft genome assembly of environmental P. fluorescens isolate EC1 consisted of 86 contigs (>500 bp), a total assembly length of 5,911,464 bp with a G+C content of 60.43%, an *N*_50_ contig size of 404 kb, and 240.4× average base coverage. The draft genome assembly of SC1 consisted of 160 contigs (>500 bp), a total sequence length of 5,963,320 bp with a G+C content of 60.42%, an *N*_50_ contig size of 390 kb, and 187× coverage.

The assembled P. fluorescens strain EC1 and SC1 genomes were annotated via the NCBI Prokaryotic Genome Annotation Pipeline (PGAP) (7) and resulted in 5,282 candidate protein-coding genes for EC1 and 5,325 candidate protein-coding genes for SC1. EC1’s genome sequence contains 10 rRNAs and 62 tRNAs, and SC1’s genome sequence contains 12 rRNAs and 66 tRNAs.

### Data availability.

This whole-genome shotgun project has been deposited at DDBJ/ENA/GenBank under the accession numbers NMRO00000000 and NNBS00000000. Raw sequencing reads are available in the NCBI Sequence Read Archive under the accession numbers SRX3001380 and SRX3001379.
